# Echoes of the Modern Training Periodization Concepts in Russia Before the October 1917 Revolution

**DOI:** 10.1519/JSC.0000000000005258

**Published:** 2025-09-29

**Authors:** Sandro Bartolomei, Gulnara Mammadova, Renato Manno, Gregory G. Haff, Jay R. Hoffman, Franco Merni

**Affiliations:** 1 Department for Life Quality Studies, University of Bologna, Bologna, Italy;; 2 Italian Olympic Committee (CONI), Rome, Italy;; 3 Strength and Power Research Group, School of Medical and Health Sciences, Edith Cowan University, Joondalup, Australia;; 4 Sport, Exercise, and Physiotherapy, University of Salford, Greater Manchester, United Kingdom; and; 5 School of Health Science, Ariel University, Ariel, Israel

**Keywords:** training history, Soviet sport, training plan

## Abstract

Bartolomei, S, Mammadova, G, Manno, R, Haff, GG, Hoffman, JR, and Merni, F. Echoes of the modern training periodization concepts in Russia before the October 1917 revolution. *J Strength Cond Res* 39(12): 1327–1330, 2025—The debate on the origins of the discipline of training periodization is still ongoing. The text “Olympic Sport,” published by B. A. Kotov in 1916, was one of the first known books to focus on training methods and strategies for performance development. The book is also considered the first to deal with training organization and periodization. In addition, Kotov was the only author to cover these topics in Russia before the Soviet Bolshevik Revolution of October 1917. Recently, our research group had the opportunity to work on the original text published in 1916 in Russian, and on the article published with the pseudonym of “Boko” in 1913 for the journal “Russky Sport.” The analysis of Kotov's articles offers an insight into periodization and training at the beginning of the 20th century. The knowledge of the historical events and of the evolution of sport allow this article to be contextualized and to understand the author's attempt to promote a different approach to athletic training. Relevant breakthroughs were included in this article, such as the use of training programs to organize long training periods, the prescription of submaximal training loads based on perceived effort and the use of specific preparation phases. In addition, the text contains one of the first notations of the concept of overtraining. Considering its novelty, “Olympic Sport” represents a revolutionary book and the author can be considered as a fundamental contributor to the development of the discipline of the training theory and a starting point of modern periodization.

## Introduction

The revival of the Olympic in 1896 marked a pivotal moment in global sporting history, catalyzing the development of organized athletics across nations worldwide (16,32). Special attention was reserved for the early Olympic Games in many countries, including Russia, which was a member of the newborn International Olympic Committee (13). Interestingly, in the first modern Olympic Games, which took place in Athens in 1896, the participation of Russian athletes was minimal. Although Russian participation in the inaugural modern Olympics in Athens was limited, the Games' revival sparked renewed interest in sport, physical education, and patriotic sentiment across numerous nations (17). This heightened engagement became particularly evident during the fifth Olympic Games in Stockholm (1912), which witnessed a significant increase in both media coverage through sport magazines and journalists, and Russian athletic participation. The Olympic Games started to become a cornerstone of a pacific interaction between the different countries in which sport was the vehicle for the “Religio Athletae” consisting in the promotion of effort, body harmony, and moral integrity (8).

The renewed interest in sport and physical performance was not a new endeavor for Russia because their first physical education program was part of the curriculum in the Moscow's School of Mathematical and Navigational Sciences in 1701 and included horse riding, fencing, sailing, games, and dance (1). The need for organized military physical training programs emerged during the Napoleonic war in 1812 and expanded after the disastrous defeat of the Russian army in the Crimean war (1853–1856) (10). In the 1840's, many Swedish instructors who specialized in military physical training were invited by the Russian government to organize and improve the physical fitness of the Russian army. Petr Henrik Ling, together with Dr. A. Georg Berglind, founded the first school for physical education teachers of the Russian army in St Petersburg, as part of the St Petersburg Academy of Medicine, and this institution operated until 1860 (1). Pyotr F. Lesgaft (1837–1909) was a Russian pedagogist, anatomist, and physician who collaborated with Dr. Berglind and developed a science-based theoretical framework for physical education in children and for research in sport science. His main book “The physical education guide for school children” was published in 1888, presenting his educational principles based on a conscious execution of the exercises, a gradual progression in exercise complexity, and an absence of physical punishment (30). Lesgaft's merits extended beyond the promotion of physical education in Russia because his work contributed to the general development of the global philosophy of physical education at the beginning of the 20th century (30,31).

Another strong Russian tradition of the 19th century was the popularity of strongmen, wrestlers, and boxers who were participating in circuses and traveling fairs operating throughout the Russian territory (31). Each of the >6,500 circuses registered in Russia in 1865 included some strongmen specializing in “heavy athletics” (20). Russian strongmen such as Georgi Hakkenschmidt became well known within the Russian territories. Other strongmen, such as Ivan Poddubny, also competed and consistently succeeded in world weightlifting championships until 1910. In addition, A. Petrov and N. Orlov won the silver medal at the 1908 Olympic games in heavyweight and lightweight wrestling, respectively (31). Regardless, the number of Russian athletes who were participating at the Olympics was still minimal relative to other delegations (e.g., 6 Russian athletes compared with 676 British athletes in 1908).

In 1913, the first Annual Russian Olympiad was organized in Kievlianin with the aim of promoting sport participation in Russia and consequently enhance performance results of Russian athletes at the Olympic Games. Although few athletes were participating at the Olympics, in 1913, >50 athletic clubs were already operating with the Russian territories (31). During this time athletics became one of the most visible events resulting in greater participation at the Olympic Games. Thus, an increased focus on the dissemination of technical knowledge about different sport disciplines and appropriate training strategies was adopted by the local federation and government with the direct goal of increasing athlete participation and performance at the Olympic Games.

The book “Olympic Sport,” published by B.A. Kotov in 1916, was the first known text to focus on training methods and strategies for running specialties, athletic jumps, and throws. The book is also considered to be the first to focus on training organization and periodization (34). In addition, Kotov is the only author to cover these topics in Russia before the Soviet Bolshevik Revolution of October 1917. The revolution deeply influenced all the aspects of society, including the role of sport and physical education and began the gradual transformation of Soviet sports into the Red Machine that excelled at the Olympic Games between 1952 and 1988.

Although the works of the Russian scientist Ivan Pavlov about conditioning and reflexes were popular at the beginning of the 20th century (28), a relationship between the conditioning process and sport training had yet to be theorized. As such, there is no reference to Pavlov's theories within Kotov's book. On the contrary, Pavlov's principles, later adopted by the communist party as foundations of Russian society, were considered as cornerstones of the periodization model later proposed by Matveev in 1958–1964 (3). In 1916, only 1 year before the Bolshevik Revolution of 1917, training and periodization were not under the influence of the Marxist–Leninist ideology that in subsequent years would dominate all the aspects of Soviet culture (31).

Recently, our research group had the opportunity to work on the original articles published by Kotov in 1916 in Russian, and on the articles published with the pseudonym of “Boko” in 1913 for the journal “Russky Sport” (4). The following points summarize the main concepts relative to training and periodization emerging by Kotov's texts.

## Training Programs as an Alternative to the Coach

Kotov supported the use of specific training plans for athletes competing in various sport disciplines. In particular, the use of training programs was strongly encouraged for novice athletes who were typically characterized by limited mastery in the training techniques required by their sport (14). Thus, these athletes required weekly and multiweek training programs, which were typically inspired by the plans used by American and Finnish coaches who were preparing their athletes for the Summer Olympic Games (14,15). Thus, it seems that during this time, the practical and theoretical knowledge about training load distribution and the organization of training sessions used by Russian specialists were based on the work of coaches from Western countries. Interestingly, the importance of training program development for experienced athletes was not a focal point of the Russian training program. It was believed that experienced athletes were an example for the younger and less experienced athletes and, according to Kotov, only required a specific organization of their training sessions and weeks during the precompetitive phase. The lack of importance placed on the training programs of advanced athletes was primarily related to the fact that these individuals, in Kotov's opinion, were aware of the principles of training, of the proper organization of the training contents, and of the correct lifestyle necessary to support performance enhancement and recovery. Another reason for the reduced need for training programs for these athletes was the constant presence of the coach to guide and monitor the athlete during each workout. In this perspective, the training program could be considered as a replacement of the coach, which may be particularly useful for novice athletes. In Russia, at the beginning of the 20th century, the presence of trainers with a specific background was very limited (31) and the Kotov's aim was to educate a new generation of coaches to promote the various disciplines and training techniques used in athletics. In other countries, such as the United States and Finland, there was greater and more consistent participation in the early Olympic games when compared with Russia. Therefore, a large number of coaches specializing in the various disciplines of athletics were already operating in sport clubs around the world. In contrast, the first athletic club appeared in Moscow only in 1909 (31).

At the turn of the 20th century, the importance for achieving good results during sport competition was attributed to a “correct” lifestyle. Several suggestions for an appropriate daily schedule, nutrition, and lifestyle were included in Kotov's books (Athletes' regime) (14,15). Although this is well understood by athletes today, Kotov's suggestions of avoiding nightlife, alcohol, and to eat meat may seem *naïve* to modern athletes, these concepts were not obvious during this time period in Russia. Interestingly, similar suggestions regarding lifestyle and training were provided to North American collegiate runners at the end of the 19th century (6,26). Kotov was probably aware of these texts, because some of the principles suggested in “Olympic Sport” were already included in some American publications (26). Thus, as stated by the author, most of the knowledge about athletic training originated by American and Finnish coaches.

After their initial presentation, these concepts were further developed in subsequent books and were combined with moral norms and communist ideology by authors from the Soviet Union and East Germany (9,23).

## Individualized and Flexible Approach to the Training Process

In “Olympic Sport,” the training process was described as a “*gradual development of human physical properties with the help of the best coach and with the help of common sense of the athlete itself*” (15). The gradual nature of the training process was considered the most appropriate long-term training strategy when attempting to elevate performance. These concepts became the foundation for a pedagogic approach to training that became central themes of the periodization concepts developed by Matveyev at the end of the 1950's (22). However, these concepts were replaced at the beginning of the 1970's with the stepwise periodization model proposed by Vorobjev and the block periodization strategies introduced by Verkhoshansky in the late 1970's (33,35,36). In these models, the loss of graduality was replaced with the use of concentrated training loads, which were introduced in the attempt to alter the individual homeostatic condition and to stimulate further adaptations in highly trained individuals (11,12,34).

The individualization of the training process was another characteristic of the approach proposed by Kotov. Kotov suggested that each sport discipline should adopt specific training strategies, and each athlete should follow different approaches to increase his performance. Although the importance of this principle was clearly stated by Kotov (14,15), the exact parameters that should be adjusted to optimize the training process were not mentioned in the Kotov's text. Interestingly, the concept of individualization is still recognized as being an important component of the training process when attempting to maximize the effectiveness of the training process. It is important to note that a key component of individualization is constant evaluation and monitoring of the technical aspects and performance by the coach (18). This is an important consideration because the training program, in Kotov's vision, should be adapted by the coach or by the athlete to the individual's needs or characteristics, resulting in what can be considered to be a flexible programming approach (15). Thus, the training process was built around the athlete, in the attempt to limit fatigue and optimize performance improvements (15). During this time period, fatigue was considered to be a negative aspect of the training process, especially when working with experienced athletes (15). Kotov suggested that the coach should be able to protect athletes against fatigue and to prevent overtraining. This seems to be one of the first notations of the concept of overtraining, which was defined in Russian with the term “Перетренированность” (pretranovarya). Kotov also suggested that if the coach was replaced by a training program, a condition that might occur with novice athletes, a high sensitivity to fatigue should be developed by the athletes to reduce the risk of overtraining (15). The sensitivity to fatigue was a persistent theme in the contemporaneous literature about training. Coaches of the recently born National College Athletic Association offered some advice to avoid “overwork” and “overexertion,” which were considered 2 of the athletes' greater dangers (26). Ten weeks of training were considered enough to be prepared for a competition and the training process should be always pleasurable to be effective (26). The alternance of training and recovery and the importance of the latter were also theorized by the great Russian physiologist Ivan Sechynov (1829–1905) who developed the theory of “active rest” that was finally published in 1903 (31).

To reduce the risk of fatigue and subsequent overtraining, Kotov suggested that sprint training should be performed at submaximal effort, using a fraction of the maximum effort (typically half or three-fourth of maximum effort). Although the first seminal studies about fatigue were conducted a few years before by Mosso (25) and Lombard (21), the scientific knowledge about central and peripheral signals involved in the perception of effort was not fully developed nor understood. In addition, the research studies that culminated in the development of perceived exertion scales and exercise-related feelings were not completed till the 1970's by the Swedish psychologist Gunnar Borg (5). However, in the early 20th century, the need for a simple and feasible method to describe exercise intensity based on perceived effort already existed in the mind of the coaches.

The flexible approach to training proposed by Kotov in an attempt to individualize the training process has been expanded by modern sport scientists (17) to include the use of athlete assessments at the beginning of each training session to better align the athletes' current state with the training program implemented on each training day. In addition, the coach may also assess the athlete's perceived recovery status at the beginning of each training session (2,19) or direct the athlete to undergo a performance assessment after the warm-up to determine the athletes' current performance capacity. These data can then be used to adjust the daily training session so that it better aligns with the athlete's current status with the intention to optimize the training process in alignment with the goals of each training phase (24). A recent investigation, however, did not support the use of subjective self-evaluations of the athlete status to improve the effectiveness of a resistance training program (2).

## The Training Period

Kotov considered training to be the single most important parameter for performance enhancement. However, at the turn of the 20th century, there was a limited number of coaches working in Russia and many athletes were forced to develop their training programs through trial and error (14,15). For that reason, Kotov's “Olympic Sport” aimed to disseminate some knowledge about the organization and structures of the training process. This book is usually mentioned as the first article presenting a division of the training period into 3 stages: general training, preparatory training, and special training. The first stage could be considered an introductory phase in which athletes were suggested to perform long walk and light gymnastic exercises at home. The second stage consisted of the period dedicated to fat mass reduction, strength improvement, and then endurance training (in this order). The last phase of training was dedicated to sport-specific training (special training) and the workouts were more focused on competitive performance. The specific phase was subdivided into 2 parts consisting of an initial part that led into what was referred to as a fundamental part. Both phases lasted 4–6 weeks in duration and contained a gradual increase in training specificity. It is important to note that Kotov's text was written during a time period where sport training was considered a hobby or something resembling artists working in the circus, and there was little scientific knowledge about the training process. However, Kotov did present a multiyear perspective on “periodization,” which, surprisingly, included sport specialization.

At the beginning of the past century, the approach to sport preparation typically consisted of the organization of a training period of 15–20 days immediately leading into a competition. Butowskij (7), in a popular manual for rowing, proposed that the optimal duration for this training period should contain the 2–3 weeks before the competition. This duration of training was recommended to avoid the monotony of a longer preparation period (5–6 weeks) and a possible reduction in motivation. Alternatively, Murphy (27) suggested that the optimal duration for the competition preparation should be approximately 6–8 weeks to reduce the negative effects of fatigue on sport technique. As time progressed, the concept of a short training period before the competition evolved into a more organized and prolonged training process. Ultimately, Kotov suggested that training and preparing for competition should be considered a full-time lifestyle. He suggested that training should focus on long-term athlete preparation, and last at least a year to develop several physical qualities in young athletes. Based on this assertion, Matveev considered Kotov to be the first author to propose a continuous training process, which also included a gradual shift from a multilateral focus to more specific training contents (23). Kotov also proposed alternating between periods of loading and recovery, which was later presented by Lauri Pihkhala (29) as a fundamental concept for sport preparation.

The analysis of Kotov's article offers an insight into periodization and training at the beginning of the 20th century. The knowledge of the historical events and of the evolution of sport allows this article to be contextualized, because the author was attempting to promote a different approach to athletic training. At the time this approach was presented, they represented significant breakthroughs, such as the use of training programs to organize long training periods and the prescription of submaximal training loads based on perceived effort. The division of the training period into 3 phases also represents a modern approach to physical preparation that characterize Kotov's article. The belief that fatigue negatively affected long-term athletic development represents a concept that became outdated by the subsequent models of periodization. Considering the novelty and the potentially misguided beliefs, “Olympic Sport” represents a revolutionary book and the author, B.A. Kotov, can be considered a fundamental contributor to the development of the discipline of training theory and a starting point of modern periodization.

**Practical Applications**

The translation of Kotov's books, published in 1916, revealed some of the main principles of training at that time and the historical contest in which training periodization was developed about a century ago. Although some of the concepts may appear quite remote nowadays, and the knowledge about training quite approximate, the book demonstrates the desire to improve performance through the organization of the training process. In particular, the need to organize the training periods in phases and the use of perceived effort to prescribe training intensity represent particularly interesting points. A better understanding of the origins of the training theory and of training periodization may improve the comprehension of the different models that were developed in the last century. In addition, coaches, athletes and sport enthusiasts may enhance the quality of their training routines through a more conscious approach to the periodization models adopted.
